# Trauma, Sepsis, and Thrombosis: Extensive Iliofemoral Deep Vein Thrombosis (DVT) Associated With Staphylococcus aureus Septic Thrombophlebitis in an Adolescent

**DOI:** 10.7759/cureus.113557

**Published:** 2026-07-28

**Authors:** Abednego Attoh, Arnold P Agyapong, Maroun Soribang Ziem, John C Ota, Henrietta D.N. Ologo

**Affiliations:** 1 Internal Medicine, Upper West Regional Hospital, Wa, GHA

**Keywords:** deep vein thrombosis, extensive deep vein thrombosis, iliofemoral vein, infection-associated thrombosis, pediatric dvt, septic dvt, septic thrombophlebitis, staphylococcus aureus septic dvt, thrombosis, traumatic vte

## Abstract

No clearly defined guidelines exist for the management of septic thrombophlebitis in the pediatric population despite its life-threatening complications. Management mainly consists of a combination of early antimicrobial therapy, anticoagulation, and source control. In this report, we present the case of a 13-year-old male who presented with a two-week history of right thigh pain and swelling, fever, chest pain, and a productive cough following trauma sustained during a football game. Doppler USG revealed extensive, near-total occlusion of the popliteal and iliofemoral veins, and blood cultures isolated *Staphylococcus aureus*. The patient was also diagnosed with pneumonia and subsequently developed septic arthritis of the right knee requiring arthrotomy and washout. Laboratory investigations were significant for leukocytosis, severe thrombocytopenia, and hepatic dysfunction consistent with sepsis. There was complete resolution of all thrombi following a prolonged course of IV antibiotics and anticoagulation, administered for 36 and 25 days, respectively. This case highlights the complex interaction between trauma, sepsis, and thrombosis. It reinforces the need to reconsider infection as a primary driver of thrombogenesis rather than a secondary complication while highlighting the associated diagnostic and therapeutic challenges.

## Introduction

Venous thromboembolism (VTE) is relatively rare in children and adolescents [1-3]. A bimodal distribution has been described, with the highest peak occurring in premature neonates, a population at increased risk of procedural interventions, particularly central venous catheterization [2]. Most cases in children occur in the presence of identifiable risk factors such as trauma, severe infection, malignancy, sickle cell disease, and inherited thrombophilia [1-4].

The major pathophysiologic mechanism of VTE involves a complex interplay among the components of Virchow’s triad, encompassing both genetic and acquired factors, with trauma contributing to this process through endothelial dysfunction [3,5]. Systemic infections and sepsis promote thrombogenesis through widespread and coordinated disruption of all three components of Virchow’s triad [6]. Endothelial injury is mediated by the release of microbial products, as demonstrated in *Staphylococcus aureus *bacteremia through the release of exotoxins, notably Panton-Valentine leukocidin (PVL) [6-9]. Both localized and systemic infections are independent risk factors for VTE and have been shown to increase the risk of thrombogenesis by up to 20-fold [10,11].

Septic thrombophlebitis, on the other hand, refers to the microbiological invasion or colonization of a thrombus. Sometimes referred to as suppurative thrombophlebitis, its etiology can be polymicrobial, with* S. aureus *being the most commonly isolated organism [12]. It may affect both superficial and deep vessels but is more commonly identified in the venous system.

Extensive deep vein thrombosis (DVT) involving the popliteal and iliofemoral veins is even more uncommon in this age group, especially in the absence of an indwelling central venous catheter, despite being associated with the highest risk of complications [13]. Data remain sparse, rendering the true epidemiologic burden of this condition substantially undercharacterized [13,14].

We present a case of extensive DVT in an adolescent following trauma, highlighting the close temporal association among trauma, venous thrombosis, *S. aureus* bacteremia, and septic arthritis. This case underscores the shared pathophysiological mechanisms, diagnostic complexity, and therapeutic challenges associated with these conditions.

## Case presentation

Patient history and examination

The patient, a 13-year-old male student, presented with a two-week history of right thigh pain, swelling, and difficulty ambulating following trauma sustained during a football match. He developed a fever one day after the trauma, followed by a productive cough with associated chest pain approximately seven days later. He initially received symptomatic treatment (IV fluids, analgesia, and supplemental oxygen), antibiotics (cefuroxime, flucloxacillin, and later clindamycin), and one unit of whole blood at a referral facility, where he was admitted for five days without significant improvement, prompting referral to our center. Associated symptoms included easy fatigability and right upper quadrant abdominal pain. There was no prior history of overt bleeding, bleeding disorders, prolonged immobilization, or any known chronic illness.

On physical examination, he appeared acutely ill and in distress. He was febrile (38.1 °C), tachycardic, mildly dehydrated, and moderately pale. Abdominal examination revealed fullness and marked tenderness in the right hypochondriac region with mild hepatomegaly. Musculoskeletal examination revealed swelling and tenderness of the right lower limb with an increased calf circumference.

Initial intervention

The patient was started on IV fluids, analgesia, and IV antibiotics (flucloxacillin and ceftriaxone) for seven days before ceftriaxone was switched to ciprofloxacin based on antimicrobial susceptibility findings. The patient also received one unit of packed red RBCs.

Investigations

Initial investigations revealed leukocytosis (WBC 13.05 × 10³/µL), anemia (Hb 8.7 g/dL), and severe thrombocytopenia (platelets 43 × 10³/µL). Blood cultures yielded *S. aureus *(>10⁵ CFU/mL), which was sensitive to ciprofloxacin and intermediately sensitive to gentamicin but resistant to ampicillin. Coagulation studies showed a prothrombin time of 10.0 seconds, an international normalized ratio of 1.03, and an activated partial thromboplastin time of 25.2 seconds, indicating no overt coagulopathy at presentation. Other laboratory investigations are summarized in Table 1.

**Table 1:** Summary of laboratory investigations. ALP, alkaline phosphatase; ALT, alanine aminotransferase; AST, aspartate aminotransferase; GGT, gamma-glutamyl transferase; HCV, hepatitis C virus

Laboratory parameter	Value	Reference range
Hepatitis B surface antigen	Negative	Negative
Hepatitis C antibody (anti-HCV)	Negative	Negative
HIV-1 and HIV-2	Negative	Negative
Hemoglobin electrophoresis	AA	AA
AST	38.4 IU/L	0-40 IU/L
ALT	44.9 IU/L	0-40 IU/L
ALP	244.4 IU/L	80-306 IU/L
GGT	235.3 IU/L	7-55 IU/L
Total bilirubin	13.03 µmol/L	0-18.8 µmol/L
Direct bilirubin	10.43 µmol/L	0-5.1 µmol/L
Albumin	20.87 g/L	37-53 g/L
Urea	9.24 mmol/L	1.7-8.33 mmol/L
Creatinine	87.5 µmol/L	50-97 µmol/L
Sodium	129.60 mmol/L	135-150 mmol/L
Potassium	4.03 mmol/L	3.5-5.5 mmol/L
Chloride	94.30 mmol/L	95-107 mmol/L

Imaging

**Figure 1:**
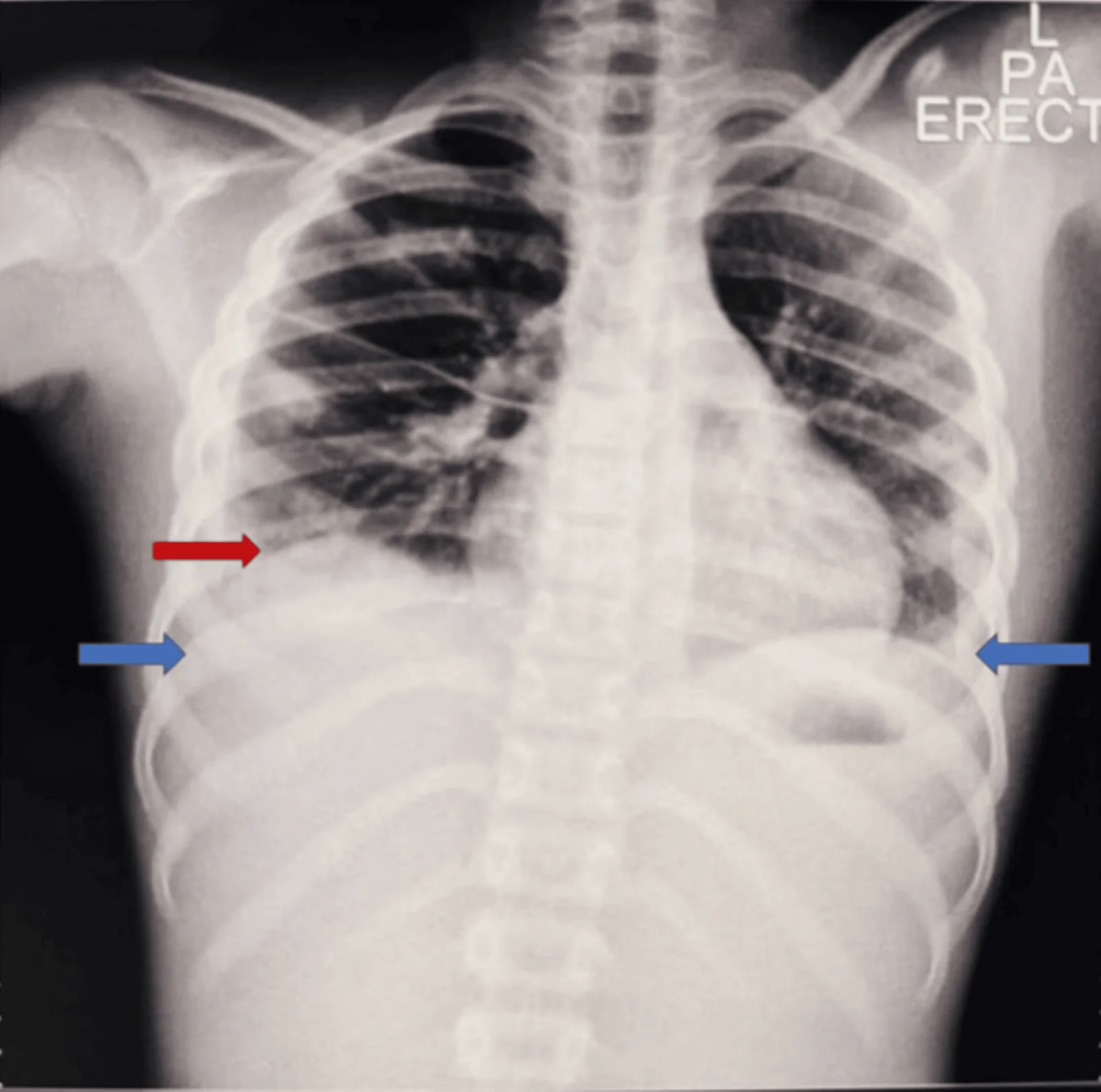
Chest radiograph showing bilateral pleural effusions (blue arrow) and right basal consolidation (red arrow), consistent with pneumonia or subsegmental atelectatic change.

**Figure 2:**
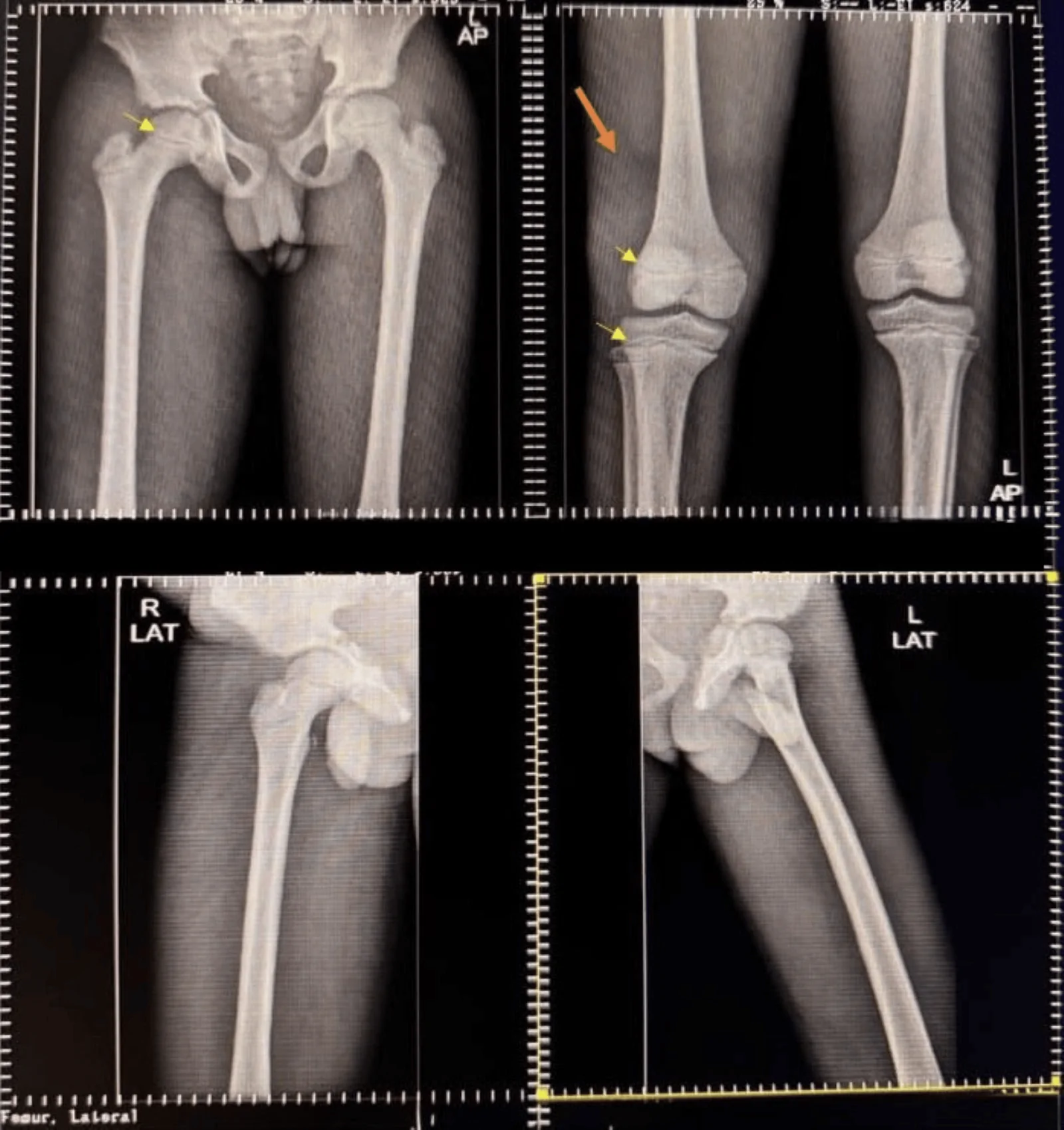
Plain radiograph of the knees and bilateral femora. Orange arrow: soft tissue swelling of the right thigh; yellow arrowhead: metaphyseal/physeal growth plates.

Doppler ultrasonography of the right lower limb, performed on day 3 of admission, revealed extensive thrombosis involving the right common femoral vein (98.8% vessel stenosis), superficial femoral vein (96.3% vessel stenosis), popliteal vein (95% vessel stenosis), and iliac veins (98.2% vessel stenosis). Abdominopelvic ultrasonography demonstrated renal parenchymal changes, inguinal lymphadenopathy, minimal ascites, and bilateral pleural effusions. The liver was, however, normal in size, measuring approximately 15.7 cm, with no focal or diffuse masses. Chest radiography showed obliteration of the costophrenic angles, suggestive of bilateral pleural effusions, with right lower lobe opacities (Figure 1). Plain radiography of the knees and bilateral femora corroborated the clinical finding of soft tissue swelling of the right thigh (Figure 2).

Clinical course and complications

A diagnosis of sepsis secondary to right lower limb cellulitis and right lobar pneumonia with parapneumonic effusion, complicated by extensive iliofemoral DVT and severe anemia, was made. A differential diagnosis of inherited thrombophilia (Factor V Leiden mutation, protein C deficiency, protein S deficiency, and antithrombin III deficiency) was considered, but screening was deferred until the acute phase had resolved because consumption of protein C and protein S during acute VTE can produce false-positive results.

On day 11 of admission, the patient’s right knee was noted to be significantly swollen and tender relative to the rest of the lower limb. It was also fluctuant and warmer than the contralateral knee. Bedside aspiration yielded 100 mL of seropurulent fluid, which was sent for microbiological studies; however, no organism was isolated, probably because of previous antibiotic administration. Ultrasonography of the right knee confirmed the presence of a joint effusion. Clindamycin was then added for improved soft tissue penetration and its anti-PVL effect, while flucloxacillin was discontinued.

By day 16 of admission, repeat testing showed worsening anemia (Hb 5.5 g/dL from 8.7 g/dL), persistent leukocytosis (WBC 18.57 × 10³/µL), and reactive thrombocytosis (platelets 470 × 10³/µL from 43 × 10³/µL), consistent with evolving sepsis and hematologic recovery, respectively. This led to the suspicion of sepsis-induced disseminated intravascular coagulation, which may account for the continued decline in hemoglobin levels without overt blood loss. The patient subsequently received three additional units of packed RBCs and underwent orthopedic surgical evaluation for possible arthrotomy and washout. On day 27, the patient developed septic shock, with blood pressure decreasing to 88/58 mmHg. An additional 200 mL of pus was aspirated from the lateral knee space and sent for microbiological studies. Antibiotic therapy was modified to include meropenem, and IV fluid boluses were administered to correct the shock, while analgesia and other supportive measures were maintained.

The patient subsequently underwent arthrotomy and washout on day 28, during which 100 mL of pus was drained from the deep posterior compartment of the knee, and a surgical drain was secured in place. Gentamicin was then added (intermediate susceptibility according to the initial blood culture report), and the patient remained stable by postoperative day 5, with drain output consistently below 10 mL. Repeat blood cultures at this stage showed no bacterial growth.

Treatment outcome

**Figure 3:**
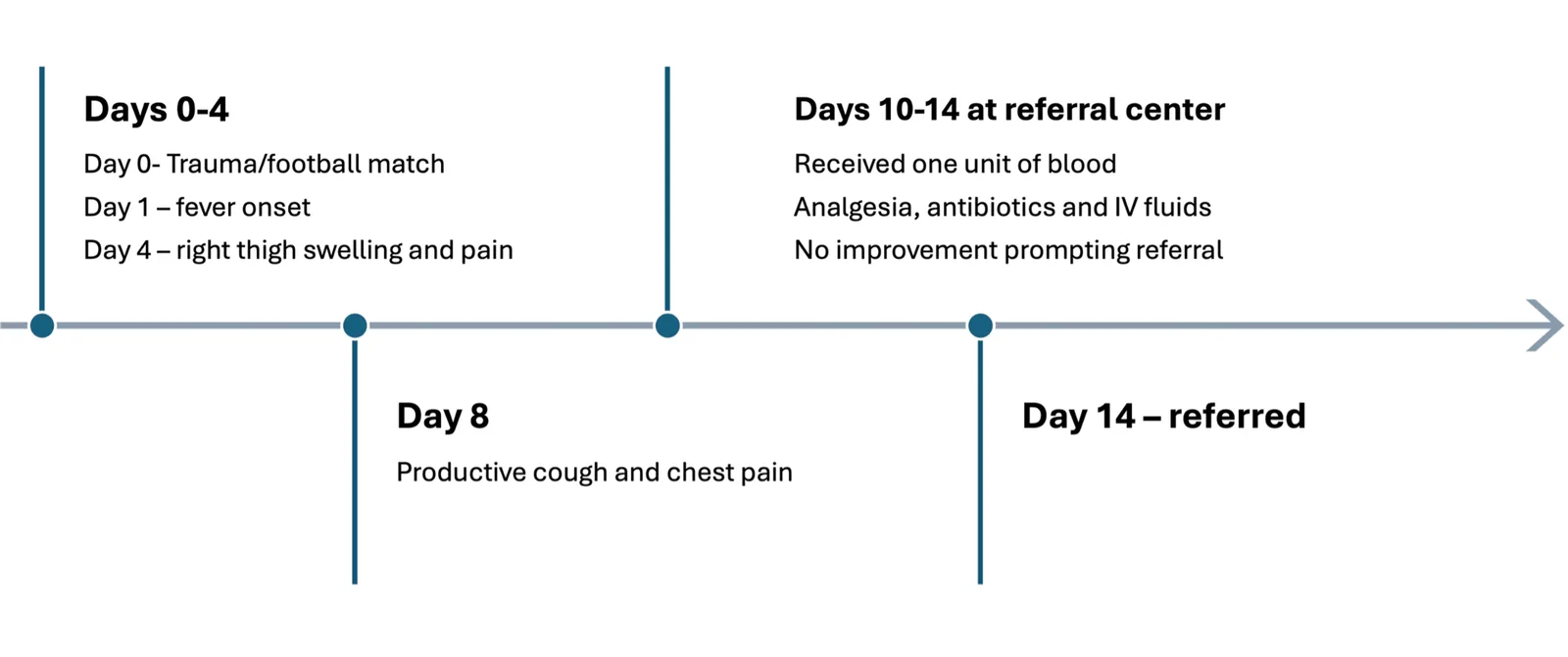
Pre-admission course (clinical timeline). This figure was created using Microsoft PowerPoint (Microsoft Corporation, Redmond, WA, USA).

**Figure 4:**
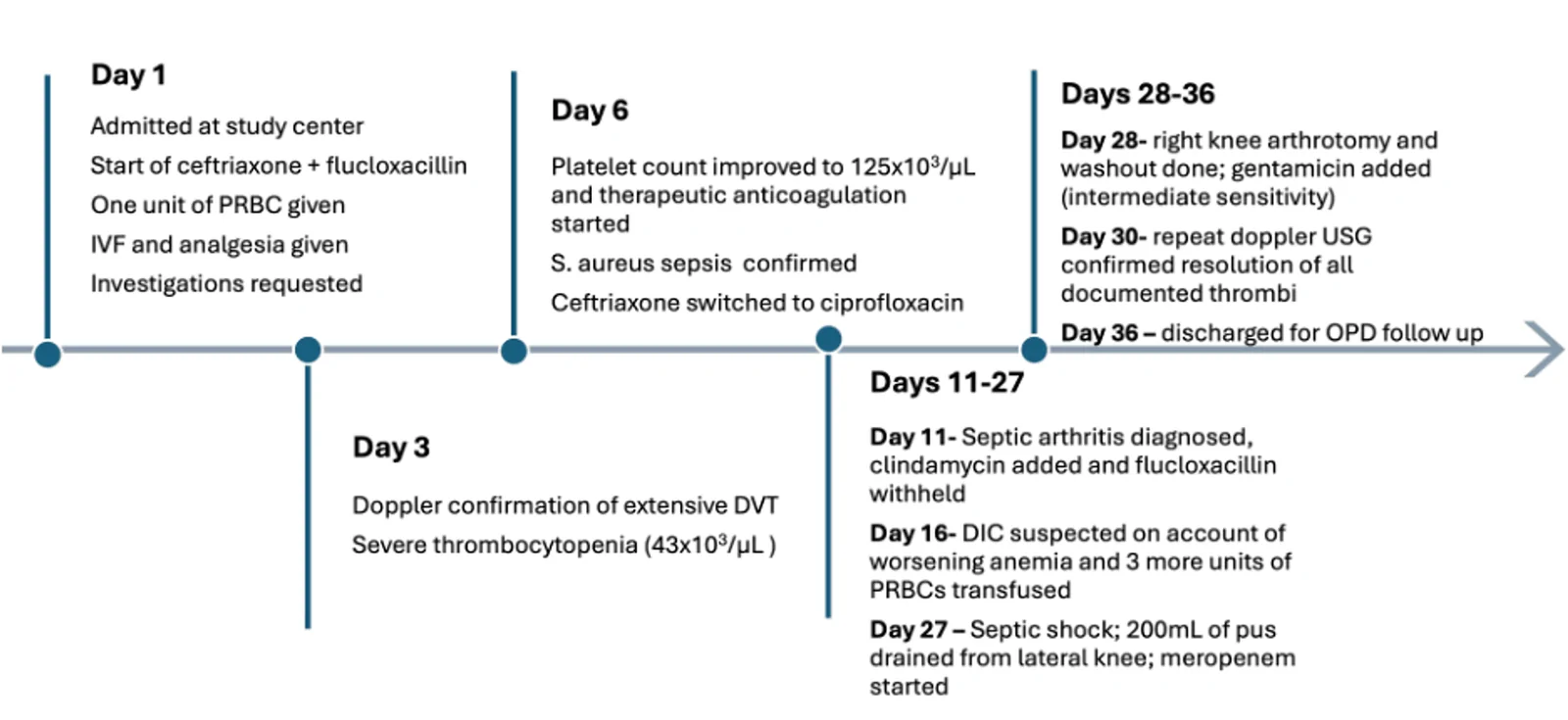
Hospital course (clinical timeline) at the study center. DIC, disseminated intravascular coagulation; DVT, deep vein thrombosis; IVF, IV fluids; PRBC, packed RBCs This figure was created using Microsoft PowerPoint (Microsoft Corporation, Redmond, WA, USA).

A total of 36 days of IV antibiotic therapy was completed during admission before discharge on oral antibiotics. The patient also responded favorably to anticoagulation during the course of admission, with clinical improvement evidenced by resolution of fever, reduction of limb swelling, and stabilization of laboratory parameters. Repeat Doppler ultrasonography of the right lower limb on day 30 showed complete resolution of all documented thrombi (Figure 3, 4).

## Discussion

VTE in adolescents remains uncommon (1.1 per 10,000 per year) compared with adults (one per 1,000 per year) [2]. Presentations involving near-total occlusion of the popliteal and iliofemoral veins in the absence of central venous catheters have been described only sporadically in the literature [15,16]. The contribution of *S. aureus* infection to thrombogenesis is thought to occur through disruption of all three components of Virchow’s triad. This process is mediated, in part, by the release of bacterial exotoxins such as PVL, which has been implicated in endothelial injury [5,6,9]. Whether PVL contributed to the thrombotic process in this case could not be determined because toxin profiling was not performed. *S. aureus *septicemia is associated with a wide range of complications, including toxic shock syndrome, septic pulmonary embolism, pneumonia, osteomyelitis, and septic arthritis.

In an institutional review of 16 pediatric patients over a five-year period, osteomyelitis was the most commonly reported complication of *S. aureus *septicemia, with methicillin-resistant *S. aureus *(MRSA) identified as the predominant isolate [17]. Notably, this patient had pneumonia and septic arthritis, which we postulate arose as direct sequelae of the underlying *S. aureus *septicemia, reflecting the organism’s propensity for hematogenous dissemination to multiple organ systems.

The central diagnostic challenge in this case was determining whether the extensive DVT represented a trauma-provoked thrombus with secondary bacterial seeding or a primary manifestation of septic thrombophlebitis. While trauma is a well-established trigger of thrombogenesis through endothelial injury, *S. aureus *infection has also been shown to initiate and propagate thrombosis through direct activation of endothelial surfaces and platelets [8]. *S. aureus *possesses procoagulant mechanisms that can lead to the formation of microthrombi and, in severe cases, multiorgan dysfunction.

The occurrence of septic arthritis within the same limb as the extensive DVT raises the possibility of either direct contiguous spread or hematogenous seeding from a common bacteremic source, favoring a septic rather than a primary thrombophilic etiology. Alternatively, the septic arthritis could have resulted from hematogenous seeding from an initial trauma-provoked DVT acting as a nidus. While this is plausible, the extent of venous occlusion and the clinical trajectory may favor infection as a primary thrombogenic agent rather than a secondary complication.

The resolution of all documented thrombi following an extended course of IV antibiotics lends further support to this interpretation, although the concurrent administration of anticoagulation precludes attributing this resolution to antimicrobial therapy alone.

Management of this case involved a combination of antimicrobial therapy, anticoagulation, and surgical source control, an approach that proved crucial to recovery. This approach is also consistent with reported management strategies for pediatric sepsis-related thrombosis, in which therapy is usually prolonged [18]. However, no consensus guidelines exist specifically for infection-associated thrombosis in children, and management remains guided by case series and extrapolation from broader pediatric VTE and adult protocols [19].

In a 10-year retrospective single-center study of 28 hospitalized pediatric patients treated for septic thrombophlebitis, the mean duration of IV and total antimicrobial therapy was 47.5 days and 65 days, respectively [18]. This compares favorably with our case, in which the patient received 36 days of IV antibiotics with a planned total treatment duration of 45 days. In the same study, the median duration of anticoagulation was 92 days, with only 57% of patients demonstrating complete resolution of thrombi on repeat imaging [18]. Notably, our patient had complete resolution of all thrombi on repeat imaging after a total anticoagulation duration of 25 days. However, that study included a larger and more heterogeneous cohort, with individualized treatment durations.

A major therapeutic dilemma encountered was the presence of severe thrombocytopenia during the acute phase, likely due to sepsis-related consumptive coagulopathy, further complicating decisions regarding anticoagulation. The anticoagulation strategy was individualized, with initial deferral because of the bleeding risk associated with severe thrombocytopenia. However, anticoagulation was carefully introduced once the platelet count began to improve (43 × 10³/µL to 125 × 10³/µL), three days after the diagnosis of DVT (day 6 of admission). This further reflects the absence of standardized protocols for anticoagulation in pediatric septic thrombophlebitis complicated by consumptive coagulopathy.

Limitations of this case include the inability to test specifically for MRSA, the absence of thrombophilia screening, meaning that inherited thrombophilia cannot be definitively excluded, and a diagnosis of septic arthritis based on grossly purulent knee aspirate rather than culture confirmation.

## Conclusions

This case highlights the multifactorial pathogenesis of VTE in the pediatric population and underscores the importance of considering infection as a plausible primary driver of thrombogenesis rather than merely an incidental comorbidity, particularly in the setting of invasive bacterial disease. This consideration is especially important in adolescents presenting with extensive thrombosis in the absence of conventional provoking factors such as central venous catheterization or systemic diseases including sickle cell disease and malignancy. Furthermore, this case draws attention to a significant evidence gap regarding the optimal duration and sequencing of anticoagulation for pediatric septic thromboembolism.
